# Supplementary figures and images for: Genome-wide analysis of long non-coding RNA expression profile in porcine circovirus 2-infected intestinal porcine epithelial cell line by RNA sequencing
Source: PeerJ. 2019 Mar 6;7:e6577. doi: 10.7717/peerj.6577 (PMC6408913; doi:10.7717/peerj.6577)

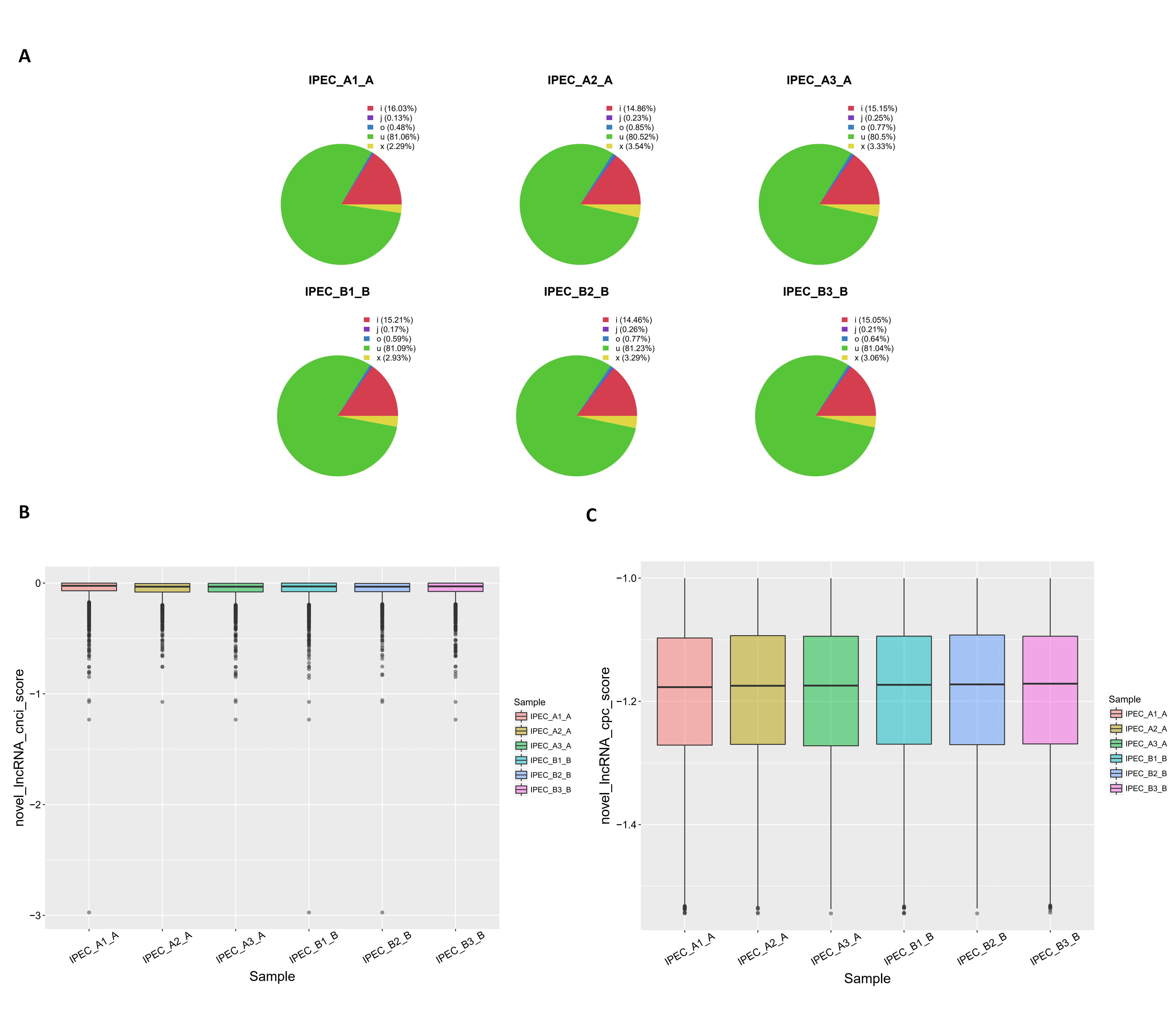

Supplement: Supplemental Information 1 — (A) Classification of raw reads. Coding potential was analyzed using CNCI (B) and CPC (C). [file peerj-07-6577-s001.png]

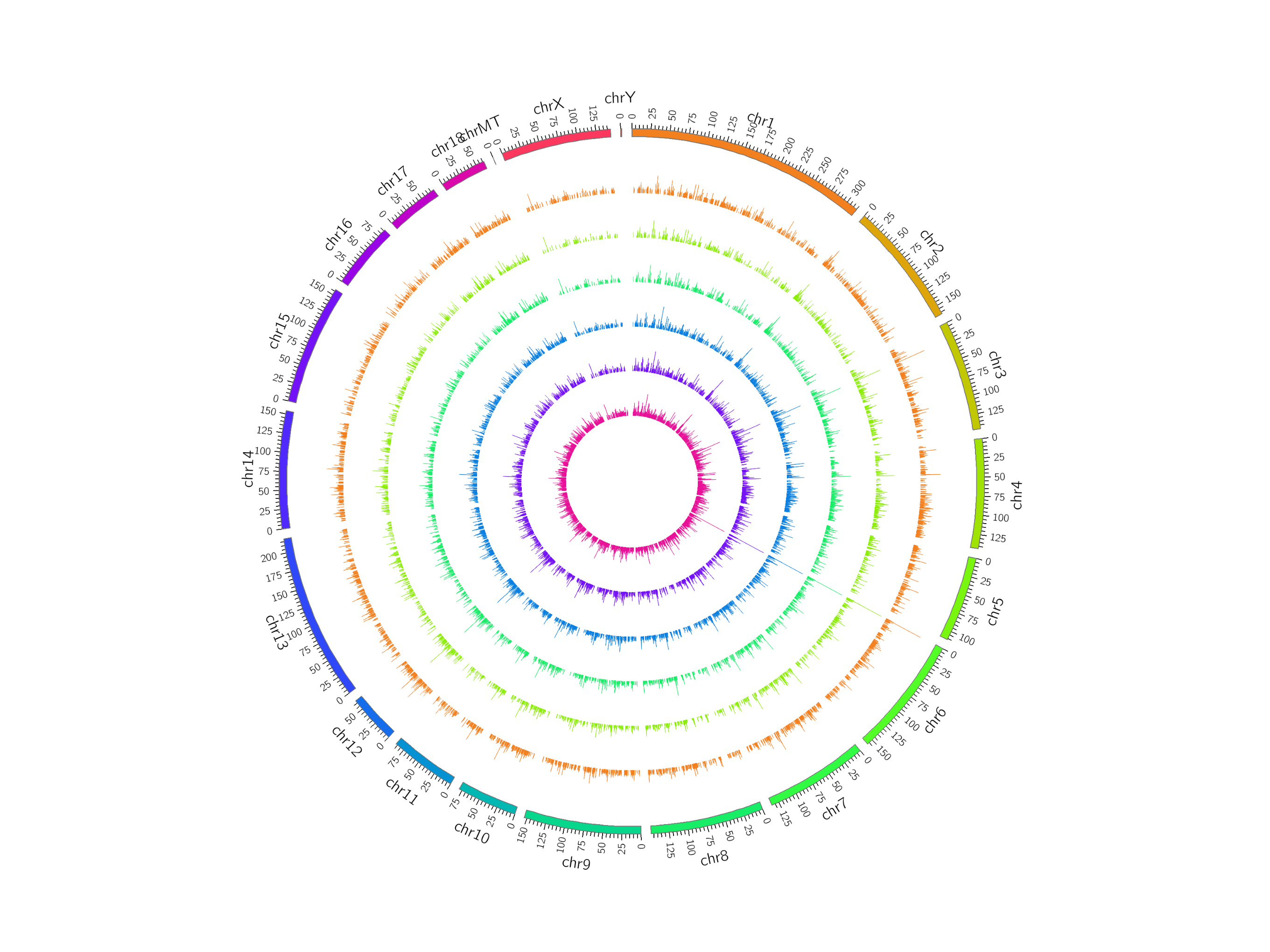

Supplement: Supplemental Information 2 — LncRNAs were broadly distributed across the chromosomes according to their locations. It comprises six concentric rings, and each corresponds to a different sample. The six different samples are IPEC_A1_A, IPEC_A2_A, IPEC_A3_A, IPEC_B1_B, IPEC_B2_B, IPEC_B3_B from outer to inner, respectively. The unit for the numbers beside each chromosome is kilobase. [file peerj-07-6577-s002.png]
